# A Novel Approach for Analyzing the Effects of Almen Intensity on the Residual Stress and Hardness of Shot-Peened (TiB + TiC)/Ti–6Al–4V Composite: Deep Learning

**DOI:** 10.3390/ma16134693

**Published:** 2023-06-29

**Authors:** Erfan Maleki, Okan Unal, Seyed Mahmoud Seyedi Sahebari, Kazem Reza Kashyzadeh

**Affiliations:** 1 Mechanical Engineering Department, Politecnico di Milano, 20156 Milan, Italy; 2 Mechanical Engineering Department, Karabuk University, 78050 Karabuk, Turkey; 3 Modern Surface Engineering Laboratory, Karabuk University, 78050 Karabuk, Turkey; 4 Department of Mechanical and Manufacturing Engineering, Ontario Tech University, Oshawa, ON L1G 0C5, Canada; 5 Department of Transport, Academy of Engineering, RUDN University, 6 Miklukho-Maklaya Street, Moscow 117198, Russia

**Keywords:** titanium matrix composites, shot peening, deep neural network, modeling

## Abstract

In the present study, the experimental data of a shot-peened (TiB + TiC)/Ti–6Al–4V composite with two volume fractions of 5 and 8% for TiB + TiC reinforcements were used to develop a neural network based on the deep learning technique. In this regard, the distributions of hardness and residual stresses through the depth of the materials as the properties affected by shot peening (SP) treatment were modeled via the deep neural network. The values of the TiB + TiC content, Almen intensity, and depth from the surface were considered as the inputs, and the corresponding measured values of the residual stresses and hardness were regarded as the outputs. In addition, the surface coverage parameter was assumed to be constant in all samples, and only changes in the Almen intensity were considered as the SP process parameter. Using the presented deep neural network (DNN) model, the distributions of hardness and residual stress from the top surface to the core material were continuously evaluated for different combinations of input parameters, including the Almen intensity of the SP process and the volume fractions of the composite reinforcements.

## 1. Introduction

Metal matrix composites (MMCs), as one of the suitable alternatives for steels, due to their good mechanical properties and light weight, have received special attention in various industries, including the automotive industry [1,2,3,4]. In the meantime, one of the most widely used MMCs in automobiles is Aluminum Matrix Composites (AMCs) [5,6,7]. For example, many scholars have studied this material for use in the manufacture of automotive steering knuckles and investigated its behavior under different working conditions [8,9]. It seems that the main reason for choosing this part is its super-critical condition in terms of failure and connection to other important parts of suspension and steering systems, which, in the case of failure, can cause serious damage to the car and its passengers [10,11]. In addition, it has been stated in some publications that this component does not have a specific shape or geometry, so it is designed based on the location of other parts of the suspension system and according to the classification of the car and its use [12]. Despite all the efforts made by researchers, Reza Kashyzadeh showed in 2023 that the use of AMCs with different percentages of titanium carbide as the reinforcement in the construction of this super-critical part is not suitable from the viewpoint of the fatigue phenomenon [13]. Moreover, he claimed that a knuckle made of this type of composite (i.e., AMC + 10, 12, and 15% TiC) works well against cyclic normal loads but, unfortunately, is not suitable against cyclic shear loads, and he finally concluded that, due to the working conditions of this component, which is subjected to non-proportional multiaxial loading due to road roughness and various maneuvers, it is better to use other MMCs. Hence, one of the suggestions is to employ Titanium Matrix Composites (TMCs). In fact, TMCs are one of the well-known metal composites and, due to their superior mechanical properties, are widely employed and considered in a variety of industries [14,15]. The mechanical properties of composites are dependent on the properties and quality of the matrix, reinforcement, and matrix–reinforcement interface that bond the formers [16,17,18]. Moreover, composites are reinforced with different types of reinforcements, such as continuous, long, and short fibers and particles [19,20,21,22]. In this regard, research achievements show that reinforced TMCs with whiskers or particles have more isotropic behaviors than reinforced ones with continuous fibers [23,24,25,26,27]. However, the whiskers of titanium monobromide (TiB) and the particles of titanium carbide (TiC) that can be obtained via high-temperature reactions [28] exhibit a high modulus, a relative chemical stability, a high thermal stability, and clean interfaces while retaining the same density and thermal expansion coefficient as TMCs [29,30,31]. In addition, TMCs that are co-reinforced by both TiB and TiC, due to their favorable properties and applications, have been investigated in many studies [32,33], and it has been proven that using these two reinforcements simultaneously has more beneficial effects than the use of either alone [34,35].

It is well known that, in metallic materials, most of the failures initiate from the surface layer [36]. Moreover, this issue is also evident in industrial parts, and the corresponding author has observed this in his previous research in the field of failure of this super-critical part, i.e., the automotive steering knuckle. Therefore, surface treatments such as SP can play a critical role in improving the mechanical properties of the surface, including the surface hardness and Compressive Residual Stress (CRS) [37,38]. It has been found by many studies that, by applying SP, the fatigue strength, corrosion, and wear resistance, as well as mechanical properties, can be improved remarkably [39,40,41]. It is clear that conducting various tests in this field is very time-consuming and expensive. However, the simulation software available on the market and the modeling algorithms provided by researchers have many errors compared to real data, which sometimes leads to catastrophic damages.

However, in recent decades, scholars have used artificial intelligence (AI) methods, such as neural networks (NNs), as a mathematical approximation solution for the prediction and analysis of complex phenomena and problems in different aspects of science and engineering [42,43]. Generally, a neural network has three main layers: input layer, hidden layer, and output layer [44,45]. As one of the first generations of neural networks, the Shallow Neural Network (SNN), which usually has one hidden layer (or a maximum of two hidden layers in some cases) that is mostly developed via the back-propagation algorithm, was used for many years, especially in the field of materials science [46,47]. For the first time in the year 2006, the presentation of the Deep Belief Network (DBN) approach by Hinton et al. [48,49] made it feasible to develop Deep Neural Networks (DNNs) based on the process of deep learning with a higher efficiency than common SNNs in prediction and analysis. Afterward, by applying major improvements in the training process of DNNs, they were used in the area of materials science [50]. Recently, in 2018 with only two papers [51,52] and in 2019 with three works [53,54,55], DNNs were employed in the area of composite materials for modeling. In the current study, a DNN model for the prediction, analysis, and optimization of a shot-peened (TiB + TiC)/Ti–6Al–4V composite was presented. In order to assess the capability of the presented DNN, the profile of the hardness and distribution of CRS on the surface and in the depth of the material, which are affected by SP, were analyzed with experimental data.

## 2. Experimental Data

**Figure 1:**
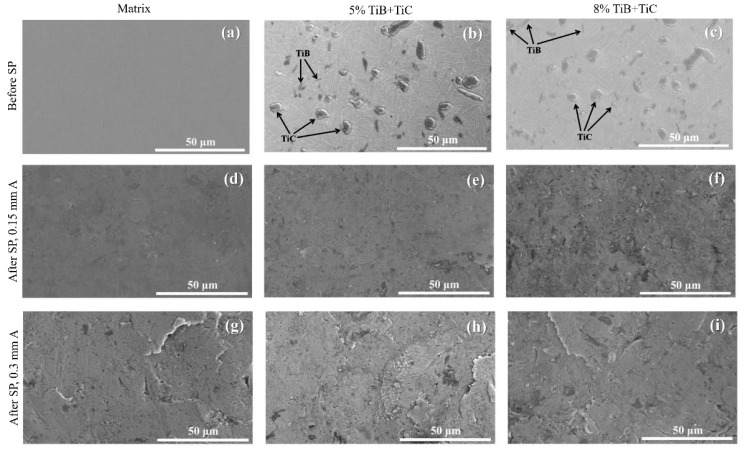
SEM images of the Ti–6Al–4V, 5% (TiB + TiC)/Ti–6Al–4V, and 8% (TiB + TiC)/Ti–6Al–4V samples (**a**–**c**) before SP, (**d**–**f**) after SP with 0.15 mm A intensity, and (**g**–**i**) after SP with 0.3 mm A intensity; adopted from [56] with permission from Elsevier.

All of the experimental data used in this study were obtained from Xie et al. [56]. Using in situ technology, composite materials of (TiB + TiC)/Ti–6Al–4V were fabricated, and two types of reinforcements (TiB + TiC) with 5 and 8% volume fractions were used. In their applied SP treatments, cast steel shots with a diameter of 0.6 mm and a hardness of 610 Hv were used, and projection pressures of 0.2 and 0.3 MPa were employed to obtain the Almen intensities of 0.15 and 0.30 mm A, respectively. In both SP treatments, 100% surface coverages were reported. In other words, only Conventional Shot Peening (CSP) treatment was carried out. Figure 1 displays scanning electron microscope (SEM) images of the prepared samples before and after applying the SP treatments. The relevant experimental results are shown in Appendix A. The results reveal that both the CRS and hardness enhance with an increase in the SP intensity, which deforms the surface layer and increases the dislocation densities. In addition, the reinforcement particles act as block sources, while the dislocation movements caused by SP have more favorable influences (see [56] for more details). An X-Ray Stress Analyzer was used to examine the residual stresses in the surface layer. The analysis was conducted using Cu-Kα radiation, with a voltage of 30 kV, a current of 25 mA, and a Ni filter. The hardness was measured using a Digital Microhardness Tester with an applied force of 2.94 N. To obtain the depth distribution of the residual stress and hardness, the thin top surface layers were sequentially removed using a chemical etching method involving a solution of water, nitric acid, and hydrofluoric acid.

## 3. Modeling and Analysis

**Figure 2:**
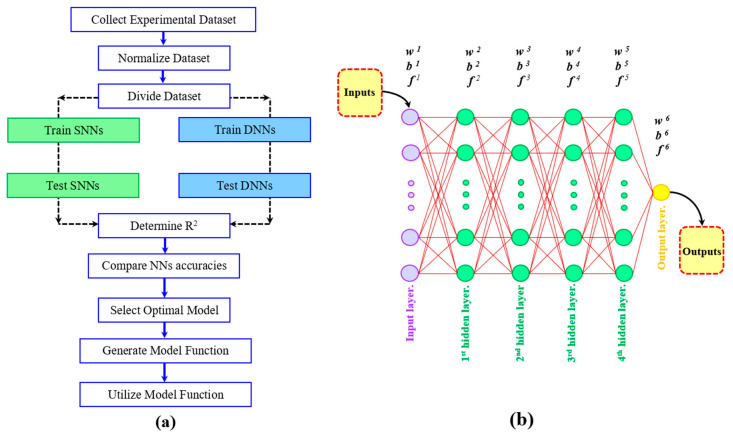
(**a**) Methodology applied in this study and (**b**) schematic illustration of DNN structure with 6 layers, including 4 hidden layers.

NNs draw inspiration from the impressive performance and abilities of the human brain in comprehending problems and providing logical solutions through functional relationships. These networks find application in the modeling and analysis of intricate and non-linear processes involving multiple variables. NNs can be utilized to model and analyze non-linear processes that involve various influential factors [57]. In order to obtain the distributions of the CRS and hardness from the top treated surface to the core material, intervals of the parameters (CRS and hardness) measured in the depth and the corresponding value of intensity were considered as the inputs, and the measured values of the CRS and hardness were specified as the outputs. The workflow and a schematic illustration of the DNN used in this study are shown in Figure 2a. Moreover, Figure 2b provides a schematic representation of the architecture of a DNN, which is essentially a modified version of an SNN with additional hidden layers. Structurally, they share many similarities. Developing a DNN may or may not involve a pre-training process. In this study, the pre-training method was not considered. In this regard, from a total of 30 samples, 24 samples were considered for the training process, and the remaining 6 samples were used for the testing step. Moreover, as a pre-process, all of the data were normalized before feeding to the network. In all of the implemented SNNs and DNNs, the value of the correlation coefficient (R^2^) was determined and considered as an accuracy factor of the NNs’ performance. The correlation coefficient calculation is presented in Appendix B.

It is well known that, in the training step of the NNs for both SNNs and DNNs, besides the hidden layer number, the number of neurons as the computational nodes has a considerable role [45,46]. The impact of the number of neurons and layers in a neural network on its modeling performance is widely recognized. The quantity of neurons serves as a significant variable parameter in the network’s structure. Increasing the number of neurons often leads to improved performance of the neural network, although it may also result in a longer computational time. In such conditions, the network development can be tuned by using a higher learning rate.

## 4. Results and Discussion

**Figure 3:**
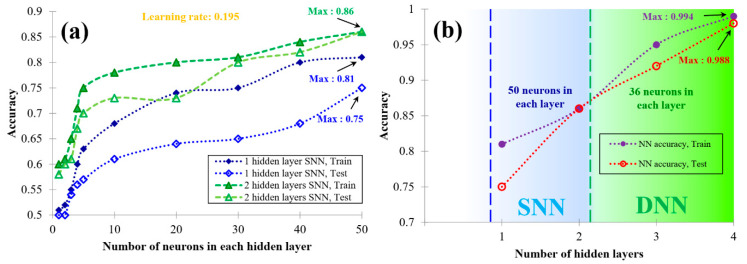
(**a**) Performance of the developed SNNs with respect to the number of used neurons and (**b**) comparison of the accuracy of NNs with different depths.

Figure 3a presents the performance of the developed SNNs with respect to the number of neurons used in each layer, and it shows that the results of the SSNs with a higher number of nodes are more accurate than the results of the others. The accuracy factor for the implemented SNN with 2 hidden layers and 50 neurons in each layer (the SNN with the highest efficiency) was determined to be 0.86, which was not favorable for further NN analysis. The role of the number of hidden layers, in both the considered SNNs and DNNs, is depicted in Figure 3b. It can be observed that, for the same data set, by enhancing the depth of the network, more accuracy can be achieved. In addition, with an equal learning rate of 0.195, to obtain an accuracy of about 0.99, the number of nodes can be decreased. All of the presented NNs were obtained via a trial-and-error approach, and among them, the DNN with an architecture of 2-36-36-36-36-2 with a tangential sigmoid activation function in the first layer and a logarithmic sigmoid transfer function in the hidden layers was chosen and employed for further analysis. In this regard, the calculation method for the corresponding model function based on the used network is presented in Appendix C. Based on the predicted results of the used network, the values of R^2^ for the training and testing processes were calculated and are presented in Table 1; the obtained accuracy values are quite acceptable, and it could be concluded that the networks were developed well.

**Figure 4:**
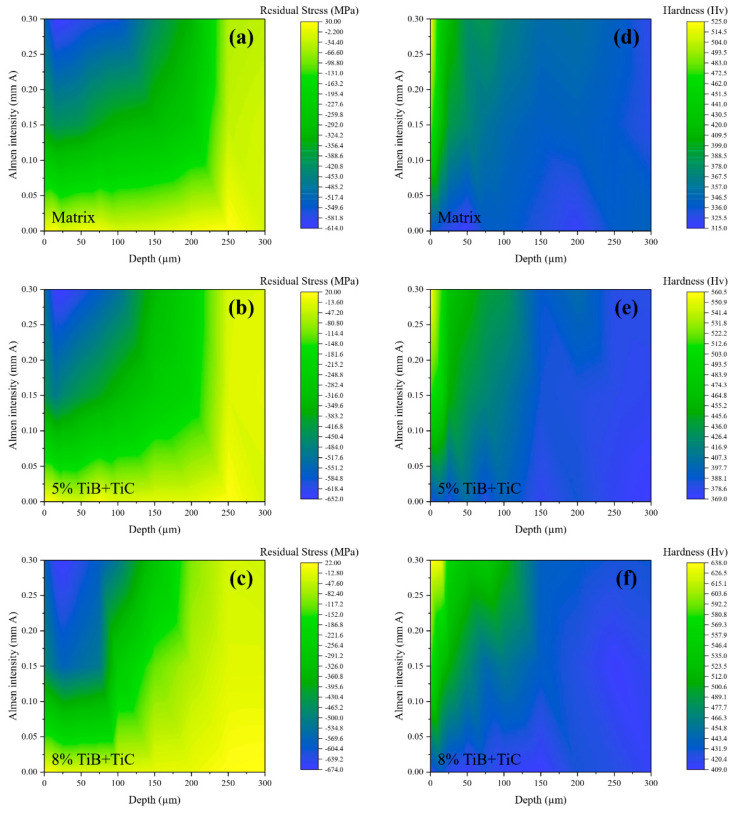
(**a**–**c**) Distribution of residual stress in (TiB + TiC)/Ti–6Al–4V composite with different reinforcement volume fractions and (**d**–**f**) profile of the hardness in the mentioned different composites.

The obtained model function of the DNN was employed to apply parametric analyses. In Figure 4, the 2D contours of the residual stress, i.e., in Figure 4a–c, and hardness, i.e., in Figure 4d–f, distributions from the top surface (depth of zero) up to a depth of 300 µm are shown. According to the used experimental data, only three values of the Almen intensity, namely, 0 (for the as-received specimens), 0.15, and 0.30 mm A, and nine depth range values were used in the network implementation; however, the DNN, as a powerful tool, continuously predicted the values of CRS and hardness for all the values of the Almen intensity in the interval of 0 to 0.30 mm A and for all the values of depth from 0 to 300 µm. Based on the predicted values, it can be observed that, by increasing the volume fraction of TiB + TiC and the SP intensity, the hardness and CRS on the surface increase. Moreover, by increasing the reinforcements, the depth of the hardness profile increases. Overall, these 2D contours can be used for an analysis of the behavior of the (TiB + TiC)/Ti–6Al–4V composite after shot peening with different intensities and a coverage of 100% and different reinforcement volume fractions.

**Figure 5:**
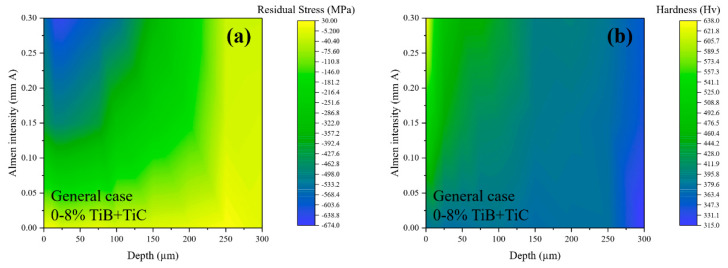
The obtained general cases for condition of 0–8% TiB + TiC in Ti–6Al–4V matrix considering the in-depth distributions of (**a**) residual stresses and (**b**) hardness in the shot-peened samples.

Considering the efficiency of the DNN for successful parametric analyses of the shot peening effective parameter of the Almen intensity on the residual stress and hardness of the treated Ti–6Al–4V and (TiB + TiC)/Ti–6Al–4V with the considered content of 0, 5, and 8% TiB + TiC (as shown in Figure 4), general cases for the in-depth distribution of residual stress and hardness were obtained for the condition of 0–8% TiB + TiC in the Ti–6Al–4V matrix, as illustrated in Figure 5, following the approach presented by Maleki et al. [58]. The results revealed that the higher the Almen intensity, the higher the in-depth inducing of CRS and hardening. Based on the predicted results, it can be seen that the highest ranges of CRS were induced by applying SP with an Almen intensity of about 0.25–0.30 mm A through a depth of about 10–55 µm below the surface. In addition, the highest values of hardness were also obtained in the same range of intensity, as well as a very thin layer of about 0–15 µm beneath the shot-peened surface. Hardening could be performed up to a depth of about 250 µm but with a lower impact.

## 5. Conclusions

In this study, the effects of variations in the Almen intensity, as one of the major process parameters of SP, on the residual stress and hardness of (TiB + TiC)/Ti–6Al–4V composites with different reinforcements volume fractions of 5 and 8% were modeled and analyzed via a DNN as a novel approach of modeling in the field of mechanical surface treatments in composite materials. According to the results, the predicted values of the DNN have an accuracy of more than 0.98% higher than the common SNNs. This study’s results can introduce the DNN as a powerful tool to analyze the variations in hardness and CRS as outputs in shot-peened TMCs by variations in the Almen intensity. The findings demonstrated a positive correlation between the Almen intensity and the depth of the induced CRS and hardening. According to the results predicted by the DNN, the most significant ranges of CRS were achieved when applying shot peening (SP) with an Almen intensity of approximately 0.25–0.30 mm A, reaching depths of around 10–55 µm below the surface. Similarly, the highest values of hardness were obtained within this intensity range, specifically within a very thin layer of approximately 0–15 µm beneath the surface that underwent shot peening. Moreover, hardening could be achieved up to a depth of approximately 250 µm, with the impact being comparatively lower in that range.

## Figures and Tables

**Table 1:** Accuracy of trained and tested DNN for each output parameter.

Output Parameter	Training Accuracy	Testing Accuracy
Residual stress	0.994 ± 0.002	0.988 ± 0.001
Hardness	0.997 ± 0.002	0.994 ± 0.004

## Data Availability

The data that support the findings of this study are available from the corresponding author upon reasonable request.

## Abbreviations

MMC	Metal Matrix Composite	AI	Artificial Intelligence
AMC	Aluminum Matrix Composite	NNs	Neural Networks
TMC	Titanium Matrix Composite	SNN	Shallow Neural Network
TiB	Titanium monobromide	DBN	Deep Belief Network
TiC	Titanium Carbide	DNN	Deep Neural Network
SP	Shot Peening	CSP	Conventional Shot Peening
CRS	Compressive Residual Stress	SEM	Scanning Electron Microscope

## Appendix A

**Table A1:** Relevant experimental results of the shot-peened (TiB + TiC)/Ti–6Al–4V [56].

Sample No.	Depth	SP Intensity (mm A)	Residual Stress (MPa)			Hardness (Hv)			Sample Type
			Matrix	5% (TIB + TIC)	8% (TIB + TIC)	Matrix	5% (TIB + TIC)	8% (TIB + TIC)	
1	0	0.00	10.42	18.04	17.93	334.72	380.37	417.57	Train
2	0	0.30	−522.83	−524.25	−575.51	524.87	560.38	637.31	Train
3	0	0.15	−375.97	−434.55	−481.94	484.31	512.07	584.43	Train
4	15	0.00	25.11	5.022	6.84	328.67	393.61	436.62	Train
5	15	0.30	−613.76	−648.62	−657.79	436.11	512.19	628.86	Train
6	15	0.15	−417.53	−499.54	−539.63	418.45	492.85	523.28	Train
7	25	0.00	−9.57	−20.26	−12.97	325.48	381.28	420.16	Test
8	25	0.30	−608.67	−650.57	−672.54	414.97	468.23	557.85	Train
9	25	0.15	−408.92	−465.67	−574.80	387.62	451.56	507.09	Train
10	50	0.00	14.35	−29.48	−9.19	315.39	403.32	411.77	Train
11	50	0.30	−581.27	−608.46	−626.77	378.62	455.55	530.79	Train
12	50	0.15	−397.63	−419.90	−545.84	372.53	431.43	475.18	Train
13	75	0.00	14.48	14.02	−12.72	338.28	381.40	423.67	Train
14	75	0.30	−564.84	−570.02	−586.49	385.38	438.64	538.40	Train
15	75	0.15	−354.00	−386.95	−516.88	358.29	420.55	446.63	Test
16	100	0.00	−12.84	−17.01	−14.41	340.03	396.67	414.43	Train
17	100	0.30	−557.53	−524.25	−515.10	368.47	433.57	509.66	Train
18	100	0.15	−324.71	−337.52	−264.28	349.94	411.35	459.31	Train
19	150	0.00	−5.26	−11.81	−8.67	330.01	375.66	409.48	Train
20	150	0.30	−422.37	−335.69	−299.08	350.72	388.76	438.64	Test
21	150	0.15	−302.74	−249.65	−120.90	340.80	386.23	435.87	Train
22	200	0.00	16.96	−13.89	−4.75	319.14	386.77	429.04	Train
23	200	0.30	−323.74	−220.36	−101.37	354.10	402.29	434.42	Test
24	200	0.15	−236.84	−194.73	−61.16	344.29	382.99	424.21	Train
25	250	0.00	28.21	14.93	17.41	337.86	375.06	424.09	Train
26	250	0.30	−46.11	−20.82	−28.14	343.11	380.31	428.50	Train
27	250	0.15	−31.80	−18.99	−15.97	336.83	378.90	410.87	Test
28	300	0.00	−13.62	−23.50	21.33	344.74	369.26	414.91	Train
29	300	0.30	−35.15	−18.99	−33.63	324.51	381.15	430.88	Train
30	300	0.15	−18.99	−11.67	−16.23	326.86	376.50	419.41	Test

## Appendix B

Coefficient of correlation (*R^2^*) can be determined using the following equation [36]:

(A1) $$R^{2}=\frac{\sum_{i=1}^{n}\left(f_{EXP,i}-F_{EXP}\right)\left(f_{ANN,i}-F_{ANN}\right)}{\sqrt{\sum_{i=1}^{n}\left({\left(f_{EXP,i}-F_{EXP}\right)}^{2}{\left(f_{ANN,i}-F_{ANN}\right)}^{2}\right)}}$$

where *n* is the number of samples used for modeling, *f_EXP_* represents the experimental value, and *f_ANN_* denotes the networks’ predicted value. The values of *F_EXP_* and *F_ANN_* are calculated as follows:

(A2a) $$F_{EXP\;}=\frac{1}{n}\;\sum_{i=1}^{n}f_{EXP,i}\;$$

(A2b) $$F_{ANN\;}=\frac{1}{n}\;\sum_{i=1}^{n}f_{ANN,i}$$

## Appendix C

Considering the optimal network, the DNN with six layers (including all of the input, hidden, and output layers), the intended model function can be determined as the following equation:

*S* (*s*(1), *s*(2)) = *a*^6^ = *f*^6^(*w*^6^*f*^5^(*w*^5^
*f*^4^(*w*^4^*f*^3^ (*w*^3^*f*^2^(*w*^2^*f*^1^(w^1^*p* + *b*^1^)+ *b*^2^) + *b*^3^)+ *b*^4^) (A3)

in which *a*^1^ to *a*^5^ are the outputs of the first to fifth layer, respectively; *a*^6^ is the sixth layer output, which is equal to the function *S* (*s*(1), *s*(2)). The functions of *s*(1) and *s*(2) illustrate CRS and hardness. Moreover, *p*, *w*, *b*, and *f* represent the inputs, weight matrixes, bias vectors, and transfer function in the layers, respectively.
